# Comparison of Myocardial Perfusion Scintigraphy and Coronary Angiography Results in Breast Cancer Patients Treated with Radiotherapy

**DOI:** 10.3390/curroncol30050346

**Published:** 2023-04-28

**Authors:** Uğur Özkan, Muhammet Gürdoğan

**Affiliations:** Department of Cardiology, School of Medicine, Trakya University, Edirne 22030, Turkey

**Keywords:** breast cancer, cardiotoxicity, cardio-oncology, coronary angiography, myocardial perfusion scintigraphy, radiotherapy

## Abstract

Breast cancer is the most common type of malignancy in women and radiotherapy (RT) is an important part of treatment. Although it reduces cancer recurrence, it has been shown to cause accerelerated athnerosclerosis. This study aimed to compare the results of myocardial perfusion scintigraphy (MPS) for ischemia investigation with coronary angiography (CAG) findings and to investigate the effect of RT on the development of coronary artery disease in breast cancer patients who underwent RT. The results of 660 patients were analyzed and compared with each other in terms of clinical, demographic, laboratory parameters and MPS results. The mean age was 57.5 years and all of them were female. When the groups were compared, the Gensini score and marking of the left anterior descending artery (LAD) area as ischemic area localization were found more, but angiographically, the rate of severe stenosis in the area indicated by MPS was found to be lower in the RT group (*p* < 0.001). While the sensitivity of MPS in the RT group was 67.5% and non-RT group was 88.5% (*p* < 0.001), the result of our study shows that the sensitivity of the MPS test is significantly lower in the patient group receiving RT.

## 1. Introduction

Breast cancer is the most common type of malignancy in women, and it is reported that one out of every 8 women is diagnosed with this type of cancer [1]. The incidence rate of female breast cancer in Turkey is 46/100,000. One in every four cancers in women is breast cancer, and 42% of breast cancer cases are in the 15–49 age group [2]. The increase in awareness about breast cancer and the developments in diagnostic methods have led to early diagnosis, and modern chemo/radiotherapy methods applied in the treatment of breast cancer, especially in the last two–three decades, have significantly reduced the mortality rates due to breast cancer, reaching a 5-year survival rate of 90% [1,3]. The mortality improvement in breast cancer patients brings with it the risk of complications called chemo/radiotherapy-related cardiotoxicity. Radiotherapy (RT) is one of the most important components of the current breast cancer treatment scheme. RT is used in 40% of cases and reduces the risk of recurrence by approximately 50% [4,5]. 

It is accepted that accelerated atherosclerosis develops in patients who underwent RT for breast cancer and irradiated the cardiac area, causing cardiotoxicity that was reported as RT-related coronary artery disease (CAD) years later [6]. Long-term follow-up studies in breast cancer patients have shown that there is a 7.4% increase per gray (Gy) within 5 years of exposure after RT, and this effect lasts for more than 20 years [7]. Because of the increased risk of CAD associated with RT, survivors of successful breast cancer treatment should be followed carefully not only before and during treatment but also in the long term after treatment, and appropriate diagnostic testing should be performed, especially for patients describing chest pain [8]. The pre-test probability of the patients is taken into account when deciding which diagnostic test to choose in daily practice. Myocardial perfusion scintigraphy (MPS) is a nuclear imaging method using Thallium-201-chloride (^201^Tl) or Technetium-99m methoxyisobutylisonitrile (Tc-99m MIBI) to measure myocardial perfusion and evaluate left ventricular function [7]. Imaging is based on the principle of the equilibrium distribution of the radionuclide in the myocardium. Thanks to its homogeneity of distribution, it can detect the localization and extent of ischemia. This makes MPS indispensable in the diagnosis and treatment management of epicardial coronary artery stenosis. However, although it is used to detect epicardial coronary artery lesions, it is known to affect this distribution in microvascular dysfunction [9]. In breast cancer patients describing chest pain with low exertion capacity and a high cardiovascular risk profile, further testing with MPS is more frequently performed than with the treadmill test to determine the possibility of CAD. However, coronary microvascular dysfunction secondary to cardiac area irradiation may reduce the reliability of MPS, especially in left breast cancer (LBC) patients. 

In this study, it was aimed to compare the results of myocardial perfusion scintigraphy for ischemia investigation with coronary angiography findings and to investigate the effect of RT on the development of CAD in breast cancer patients who underwent RT.

## 2. Method

### 2.1. Study Population

The study was performed in a single center and with a retrospective design. The study included 165 female patients who underwent coronary angiography (CAG) because of MPS positivity in the follow up from the patients who were diagnosed with LBC pathologically between 2007–2021 and underwent RT in their treatment. As the control group, 495 female patients who did not have a history of breast cancer or RT and underwent CAG due to MPS positivity in the same period were included in the study. MPS testing was performed because patients described cardiac symptoms. Patients with known cardiovascular disease, patients with left bundle branch block or conduction defects, patients with segmental wall motion defects, patients with ejection fraction (EF) <40%, patients with a history of cardiac surgery, patients with advanced valve pathology, patients with cardiac tumors and patients with breast prosthesis were not included in the study. In addition, male patients (only 1 patient) and those patients receiving adjuvant and neoadjuvant chemotherapy were excluded to ensure patient and data standardization and to exclude chemotherapy-related cardiotoxicity. To determine the effect of RT, patients with hypertensive heart diseases, infiltrative heart diseases, and advanced aortic stenosis, which were shown to cause microvascular coronary dysfunction in the literature, were excluded from the study. All female patients who met the inclusion and exclusion criteria and underwent CAG in our center for MPS positivity between 2007 and 2021 were included in our study. All things considered, ≥80% stenosis in the indicated ischemia region (≥50 stenosis in the left main coronary artery (LMCA)) as a result of CAG was accepted as MPS true positivity. Strictures between 60 and 80% were evaluated according to the fractional flow reserve (FFR), and lesions with an FFR value of <0.80 were considered critical, and the MPS result of these patients was recorded as true positive [10,11]. Angiographically, LMCA lesions with a rate of 40–50% were evaluated by intravascular ultrasonography according to the decision of the cardiology-cardiovascular surgery council. In patients with other CAG results, the MPS result was considered a false positive.

Our study was approved by the Trakya University Medical Faculty Ethics Committee (TUTF-GOBAEK 2021/476) and complied with the Helsinki Declaration.

### 2.2. Clinical Data Collection

Socio-demographic, pre-MPS laboratory results, echocardiographic, angiographic, and RT and cardiac area radiation exposure data of 660 patients included in the study were recorded from the hospital automation system. CAG and/or PCI indications of the patients were determined within the framework of current cardiology guidelines based on the history, ECG, cardiac biomarkers, and non-invasive tests [10,11]. Accordingly, CAG is applied to patients with an ischemic area >10% in the left ventricle in MPS. CAG is performed using the femoral or radial route, depending on the operator’s preference, and the results are evaluated by a council of at least 2 cardiologists or a cardiologist and a cardiovascular surgeon. Reperfusion strategy is determined by this council according to current guidelines. For our study, CAG images were examined by 2 cardiologists, and data were recorded in terms of lesion severity and extent. Lesion severity and extent were assessed using the Gensini scoring system. Gensini score (GS) is an angiographic scoring system that is used to quantify and characterize the complexity of CAD. It is calculated for each lesion by considering severity score, lesion zone, and coronary collateral adjustment factor. The final score is obtained by summing each coronary lesion score [12].

### 2.3. Statistical Analysis

The statistical analysis of the data was conducted using version 26.0 of the SPSS software (SPSS Inc, Chicago, IL, USA). The normal distribution of the data was evaluated using the Kolmogorov–Smirnov test. Normally distributed descriptive data are indicated in the tables as mean + standard deviation and non-normally distributed descriptive data are given in the tables as median (min-max). The independent sample *t*-test (for normally distributed data) or the Mann–Whitney U test (for non-normally distributed data) was used in the analysis to compare quantitative data. The Chi-square test was used to compare categorical data. Categorical data analysis is indicated by numbers and percentages in the tables. The effect of variables on cardiovascular disease was measured using univariate analysis. Values with *p* < 0.05 statistical significance in univariate analysis were used for multivariate analysis modeling. Cox regression analysis was used to model the risk factors affecting the development of coronary artery disease and Kaplan–Meier analysis was used to calculate the estimated time.

## 3. Results

### 3.1. Clinical Characteristics

Among the 660 participants included in the final analysis, the median age was 57.5 years (36–85) and all of them were female. Socio-demographic characteristics, medical histories, and laboratory and angiographic data of the patients are shown in Table 1. When the demographic data of the groups were compared, the mean age of the group receiving RT was 56.5, which was lower than the control group (*p* = 0.036). Again, the body mass index of this group was lower at 25 kg/m^2^ ( *p* < 0.001). There was no significant difference between the groups in terms of other CAD risk factors. When the medication histories of the patients before angiography were compared, ASA use was higher in the group that did not receive RT (*p* < 0.001). There was no significant difference for other medications.

When the laboratory data of the groups were compared, HDL, triglyceride, albumin, and hemoglobin values were found to be lower in the RT group than in the control group (*p* < 0.001). The creatinine value was found to be lower in the group that did not receive RT (*p* = 0.036). There was no significant difference between other laboratory parameters. When the groups were compared in terms of coronary angiography data, the Gensini score of the patients who received RT was found to be significantly higher (*p* < 0.001). Marking of the LAD area as ischemic area localization was found more in MPS in the RT group (*p* < 0.001). In the MPS results of the control group, RCA and Cx fields were higher in ischemic area localization. Angiographically, the rate of severe stenosis in the area indicated by MPS was found to be lower in the RT group (*p* < 0.001). Among patients in the RT group who underwent CAG, 32.5% had epicardial coronary artery stenosis of 60% or less. This rate was 11.5% in the control group. While the sensitivity of MPS in the RT group was 67.5%, the sensitivity of MPS in the group that did not receive RT was 88.5%. The difference was statistically significant (*p* < 0.001). Statistically significant differences were not found in MPS results in relation to cancer stages (*p* > 0.05, it is shown with the letter system in Table 1).

### 3.2. Analysis of RT Regimen

In the RT group, the mean time to coronary angiography after RT was 41 months. Data for the RT dose regimen are shown in Table 2. There is no statistically significant difference in terms of cancer stages and RT dose regimen.

### 3.3. Cox Regression Analysis Results

The results of the Cox regression analysis of the risk factors affecting the development of CAD are shown in Table 3. Accordingly, in the univariate analysis, DM, LDL, and total cholesterol levels, which are traditional risk factors, were found to be significant risk factors for the development of CAD, while in the multivariate analysis, LDL cholesterol (*p* < 0.001) and total cholesterol value (*p* = 0.002) were found to have a significant effect. The data of the Cox regression analysis results of the RT group are shown in Table 4. 

Accordingly, in the univariate analysis, in addition to the presence of DM, LDL level, total cholesterol level, and smoking, which are among the traditional risk factors, the mean heart dose exposed as a result of cardiac area irradiation, mean LAD dose, LAD proximal mid-distal doses were also found to be significant (*p* < 0.001, for each). In the multivariate analysis, it was found that the most important factor affecting the development of CAD was the dose of RT to which the coronary arteries were exposed.

### 3.4. Kaplan–Meier Analysis Results

Kaplan–Meier analysis results of the group receiving RT are shown in Table 5. According to the analysis, when the effects of traditional risk factors and levels on the time to CAD development were expressed in months, it was determined that after RT exposure, the LDL level was above 130 mg/dL, the total cholesterol level was above 190 mg/dL, and the presence of DM significantly accelerated the development of coronary atherosclerosis.

## 4. Discussion

The most important findings of the study can be listed as follows: (1) the sensitivity of the MPS test performed for angina examination in patients who were exposed to cardiac area irradiation due to left breast cancer (who received RT) decreased significantly; (2) coronary artery disease is both earlier and more common in the group receiving RT due to accelerated atherosclerosis; (3) it is unreliable to investigate cardiac ischemia with MPS in patients with RT exposure to the cardiac field who develop anginal symptoms (whether or not the developing cardiovascular disease is secondary to RT); and (4) although a correlation was found in univariate analysis between traditional risk factors and the development of coronary artery disease in patients receiving RT, the results of the multivariate analysis show that the strongest relationship is correlated with the mean heart dose received. To our knowledge, this is the first study to evaluate the sensitivity of MPS, a non-invasive examination method, in patients exposed to cardiac area irradiation and describing anginal symptoms.

Although there are studies in the literature showing the development of RT-related myocardial damage, the correlation of MPS performed for angina examination and coronary angiography data in these patients was not investigated, and the sensitivity of MPS for this patient group was not investigated [13,14,15,16]. MPS is one of the non-invasive tests recommended by current cardiology guidelines for the preliminary diagnosis of coronary artery disease in patients who do not have acute coronary syndrome but describe angina in daily practice [9,10]. 

In addition to being relatively easily accessible, MPS is a very reliable examination test with high sensitivity rates. Its sensitivity in detecting severe CAD is in the range of 85% to 98%, making it one of the main imaging modalities for CAD examination [17,18,19]. However, it has also been shown that several causes such as cardiomyopathies and cardiac tumors, especially conduction defects and soft tissue attenuation, reduce the specificity of MPS [20,21]. In our study, the sensitivity of MPS was found to be 88.5% in patients in the control group. This result is compatible with the literature.

Although the tissue attenuation rate was much lower in the group receiving RT, due to the previous mastectomy, the sensitivity rate of MPS was 67.5%. This value was found to be significantly lower than the control group. RT is a treatment modality that targets cell death in tissue using megavoltage photon beams [22]. Fibrosis occurs as a result of the accumulation of matrix proteins and collagens in the extracellular area of the tissue exposed to radiation as a result of RT. This effect, which occurs in a dose-dependent manner, causes angina in patients due to dysfunction in the microvascular bed [23,24]. In the literature, it has been shown that endothelium-dependent vasoreactivity is impaired long before the fibrosis stage occurs [25]. This is manifested as a decrease in myocardial perfusion reserve and heterogeneous distribution of radiotracers [26,27]. 

Radiation exposure occurs in the LAD coronary artery area as a result of the irradiation of the internal mammary chain in left breast cancer patients undergoing RT [28]. In our study, in the MPS results of the group receiving RT, it was observed that the ischemic area was predominantly localized to the LAD area and increased the Gensini score due to increased plaque load in this area. This situation is compatible with the existing information in the literature [29]. Our study was designed with patients describing their symptoms. In this patient group, it was observed that the symptoms caused by microvascular damage secondary to RT started before the plaque load in the epicardial coronary artery reached a significant rate. For this reason, it was observed that the rate of severe CAD (60% or more with stenosis) was found to be lower with coronary angiography in this area. This may explain why MPS sensitivity was found to be lower in the RT group. It has been shown that RT exposure increases oxidative stress in tissue, causing endothelial dysfunction and an increase in pro-inflammatory cytokines [30]. This may explain the pathophysiological mechanism of diffuse accelerated atherosclerosis associated with RT. The fact that the Gensini score, which indicates the prevalence of coronary artery disease, was higher in the RT group in our study, can be interpreted as a result of the pathophysiological mechanism mentioned.

In an intracoronary ultrasonography study in which patients who underwent CAG and were found to be normal due to ischemia detected in MPS in the literature, it was shown that conventional angiography was insufficient in detecting atheroma plaque burden [31]. It was concluded that the ischemia detected in MPS may be due to the plaque load not seen in angiography. There are data in the literature showing that there is a relationship between the presence of uncontrolled hypertension (HT) and familial hypercholesterolemia and false positive MPS results [32]. In our study, there was no difference between secondary causes such as HT and hyperlipidemia (HL), which may lead to a lower detection of MPS sensitivity in the RT group. It is known that DM, HL, smoking, and age are common risk factors for cancer and cardiovascular diseases. In our study, in addition to these risk factors, the relationship between RT cardiac area irradiation and the development of atherosclerosis was found to be compatible. These findings are consistent with data in the literature [33]. 

In our study, it was observed that the occurrence of cardiac symptoms in the RT group developed earlier than in the control group and started 41 ± 6.9 months, on average, after exposure. This finding suggests that RT exposure triggers anginal symptoms much earlier by causing a synergistic interaction with both direct and traditional risk factors. Therefore, it can be emphasized that in breast cancer patients scheduled for RT, modifying traditional risk factors to prevent possible cardiotoxicity plays a key role in successfully continuing cancer treatment [34,35,36]. 

In our study, we found that the mean heart dose increased the development of stenosis in the epicardial coronary arteries by 2.015 (95% CI: 1.429% to 2.841%) times. The data that the most important factor affecting the development of coronary artery disease in RT patients is the exposed heart dose once again shows the necessity of heart-protective modern RT applications in breast cancer patients. While a relationship was found between the mean LAD dose, mid LAD, distal LAD doses, and the development of coronary artery disease, the same relationship was not found with the proximal LAD dose. It has been reported in the literature that it is sensitive to atherosclerotic lesions triggered by RT in the proximal coronary arteries [37]. The reason why RT exposure in proximal LAD did not reach statistical significance in the multivariate analysis in our study may be related to the RT modeling applied to breast cancer patients in our center.

The main limitations of our study can be listed as follows. First of all, our study was conducted in a single center and with a retrospective design. The patient group who received RT and was found to be normal in MPS was not represented in the study. Therefore, although the sensitivity of the test was found to be low, its specificity could not be determined. Patients who received chemotherapy were excluded from the study for study standardization. Although it is possible to see the true effect of RT only in this way, we do not know the results for the patient population receiving chemo/radiotherapy.

## 5. Conclusions

Our study found that the sensitivity of the MPS test was significantly lower in the RT group, which is valuable in terms of showing that coronary angiography performed according to the MPS test result or reperfusion strategy decisions to be taken in this direction may be incorrect.

It is seen that RT exposure has a synergistic effect with both direct and traditional risk factors and causes cardiac symptoms to be seen in earlier periods. Our study reveals once again the importance of minimizing RT exposure and controlling and treating traditional risk factors for cardiovascular diseases in the prevention of possible cardiotoxicity in breast cancer patients scheduled for RT. Although our study was limited to breast cancer patients, it is valuable in that it shows that different diagnostic tests (such as coronary CT) should be considered, in addition to MPS in demonstrating epicardial coronary artery stenosis in patients describing anginal symptoms exposed to cardiac area irradiation. Prospective randomized controlled studies are needed, especially in patients receiving isolated cardiotoxic chemotherapy and patients receiving combined therapy (RT and CT). In addition, in the future, the comparison of coronary CT angiography, MPS and fractional flow reserve-supported results will be more useful in determining the non-invasive imaging method to be chosen.

## Figures and Tables

**Table 1:** Patient and treatment characteristics.

Characteristics	RT Group	Non-RT Group	*p*
	(*n* = 165)	(*n* = 495)	
Demographic parameters			
Age (years)	56.5 ± 7.1	58.6 ± 10	0.036
BMI (kg/m^2^)	25.0 ± 2.4	28.1 ± 1.7	<0.001
HT, *n* (%)	94 (57)	311 (62.8)	0.26
DM, *n* (%)	35 (21.2)	147 (29.6)	0.07
Stroke, *n* (%)	8 (4.8)	33 (6.6)	0.63
Smoking, *n* (%)	25 (15.2)	102 (20.6)	0.86
Drinking, *n* (%)	29 (17.6)	111 (22.4)	0.27
Familial history, *n* (%)	16 (9.7)	75 (15.1)	0.182
ASA, *n* (%)	34 (20.6)	204 (41.2)	<0.001
Statin, *n* (%)	53 (32.1)	108 (21.8)	0.35
β Blocker, *n* (%)	66 (40)	162 (32.7)	0.17
Left ventricular ejection fraction (%)	51.5 ± 5.4	52.1 ± 6.3	0.28
Laboratory parameters			
HDL (mg/dL)	31.7 ± 7.6	37.3 ± 5	<0.001
LDL (mg/dL)	133.2 ± 24.5	138.6 ± 32.1	0.09
Triglycerides (mg/dL)	179.5 ± 33.7	202.6 ± 29.2	<0.001
Total cholesterol (mg/dL)	204.9 ± 35.4	199.1 ± 40.9	0.173
Creatinine (mg/dL)	1 (0.59–1.6)	0.9 (0.49–1.7)	0.036
Albumin (g/dL)	3.5 (3.4–3.6)	3.7 (3.6–3.8)	<0.001
Hb (g/dL)	11.9 (11.2–12.7)	12.8 (11.9–13.7)	<0.001
PLT (10^3^/µL)	257.8 ± 72.2	260.9 ± 74	0.7
Lym (10^3^/µL)	2.2 (1.5–2.6)	2 (1.5–2.3)	0.29
WBC (10^3^/µL)	7.9 ± 1.9	8.2 ± 1.5	0.08
Angiographic parameters			
Gensini	38 (0–69)	32 (7–45)	<0.001
Ischemic field			
LAD, *n* (%)	79 (47.9) ^a^	141 (28.5) ^b^	<0.001
LCX, *n* (%)	47 (28.5) ^a^	159 (32.1) ^b^	
RCA, *n* (%)	39 (23.6) ^a^	195 (39.4) ^b^	
Ischemic field stenosis degree			
0–20% stenosis, *n* (%)	17 (10.3)	30 (6.1)	<0.001
20–40% stenosis, *n* (%)	28 (17)	21 (4.2)	
40–60% stenosis, *n* (%)	8 (4.8)	6 (1.2)	
60–80% stenosis, *n* (%)	105 (63.6)	390 (78.8)	
80–100% stenosis, *n* (%)	7 (4.2)	48 (9.7)	
MPS sensitivity	67.50%	88.50%	<0.001
Radiotherapy technique			
IMRT	140 (84.8)		
VMAT	13 (7.9)		
WBRT	12 (7.2)		
Breast cancer stage			
Stage 0 (DCIS) Tis	38 (23)		
Stage 1 T1N0M0	53 (32.1)		
Stage 2A			
T0N1M0	2 (1.2)		
T1N1M0	5 (3)		
T2N0M0	14 (8.4)		
Stage 2B			
T2N1M0	17 (10.3)		
T3N0M0	36 (21.8)		
Stage 3			
3A	0		
3B	0		
3C	0		
Stage 4	0		

Values are mean ± SD, *n* (%), or median (IQR). ^a, b^ = There is no significant difference between groups with the same letter. Abbreviations: ASA, acetylsalicylic acid; BMI, body mass index; DM, diabetes mellitus; DCIS, ductal carcinoma in situ; Hb, hemoglobin; HDL, high-density lipoprotein; HT, hypertension; IMRT, intensity-modulated radiation therapy; ischemic field stenosis degree, the degree of stenosis detected angiographically in the area where ischemia is indicated on myocardial nuclear imaging; LAD, left anterior descending artery; LCX, left circumflex artery; LDL, low-density lipoprotein; Lym, lymphocyte; MPS, myocardial perfusion scintigraphy; RCA, right coronary artery; Tis, carcinoma in situ; VMAT, volumetric-modulated arc therapy; WBC, white blood count; WBRT, whole breast radiation theraphy.

**Table 2:** Heart and coronary artery doses from breast radiotherapy regimens *.

	RT-CAG Time	Mean Heart Dose	Mean LAD Dose	Mean LAD Prox Dose	Mean LAD Mid Dose	Mean LAD Distal Dose
Total	41 ± 6.9	2.6 ± 0.1	5.9 (4.7–7.1)	3.1 (2.5–3.7)	8.1 (7.3–9.5)	8.7 ± 1.7
DCIS	43 ± 4.1 ^a^	2.5 ± 0.2 ^a^	6.1 (4.7–6.9) ^a^	2. 9 (2.8–3.4) ^a^	7.7 (7.4–8.5) ^a^	8.5 ± 1.9 ^a^
NLA, NNBC	39 ± 5.3 ^a^	2.6 ± 0.1 ^a^	5.7 (4.9–7.1) ^a^	3.1 (2.8–3.7) ^a^	8.1 (7.3–8.9) ^a^	8.8 ± 1.6 ^a^
NLA, NPBC	41 ± 5.7 ^a^	2.6 ± 0.1 ^a^	5.9 (5.2–7) ^a^	3.2 (2.5–3.5) ^a^	8.3 (7.5–9.5) ^a^	8.7 ± 1.5 ^a^

Values are mean SD, *n* (%), or median (Q1–Q3). * The dose unit was calculated in gray. ^a^ = There is no significant difference between groups with the same letter. Abbreviations: DCIS, ductal carcinoma in situ; LAD, left anterior descending artery; NLA, NNBC, non-locally advanced, node-negative breast cancer; NLA, NPBC, non-locally advanced, node-positive breast cancer; RT-CAG time, time from initiation of RT to coronary angiography, months.

**Table 3:** COX regression analysis results.

	Univariate		Multivariate	
	HR (95% CI)	*p* *	HR (95 %CI)	*p* ɫ
Age	1.014 (0.987–1.042)	0.319	-	-
HT	0.934 (0.642–1.36)	0.723	-	-
DM	2.12 (1.394–3.225)	<0.001	1.362 (0.884–2.098)	0.161
Stroke	0.561 (0.178–1.771)	0.324	-	-
Smoking	1.619 (0.994–2.637)	0.053	-	-
Drinking	0.873 (0.537–1.42)	0.584	-	-
Familial history	0.658 (0.319–1.36)	0.259	-	-
BMI	0.996 (0.92–1.077)	0.917	-	-
HDL	1.002 (0.978–1.026)	0.876	-	-
LDL	0.991 (0.984–0.999)	0.023	1.022 (1.013–1.032)	<0.001
Triglycerides	0.998 (0.992–1.004)	0.469	-	-
Total cholesterol	0.993 (0.988–0.999)	0.026	1.008 (1.003–1.014)	0.002
Creatinine	1.015 (0.474–2.173)	0.969	-	-

* Calculated using the enter method. ɫ Calculated using the backward Wald method. Abbreviations: BMI, body mass index; DM, diabetes mellitus; HDL, high-density lipoprotein; HT, hypertension; LDL, low-density lipoprotein.

**Table 4:** Examination of risk factors affecting coronary artery disease in the group exposed to cardiac field irradiation (Cox regression univariate and multivariate results).

	Univarite Analiz		Multivariete Analiz	
	HR (95% CI)	*p* *	HR (95% CI)	*p* ɫ
Age	1.025 (0.996–1.055)	0.09	-	ns
HT	0.934 (0.642–1.36)	0.72	-	ns
DM	2.12 (1.394–3.225)	<0.001	-	ns
Stroke	0.561 (0.178–1.771)	0.32	-	ns
Smoking	1.619 (0.994–2.637)	0.05	-	ns
Drinking	0.873 (0.537–1.42)	0.58	-	ns
Familial history	0.658 (0.319–1.36)	0.26	-	ns
BMI	0.996 (0.92–1.077)	0.92	-	ns
HDL	1.002 (0.978–1.026)	0.88	-	ns
LDL	0.991 (0.984–0.999)	0.02	-	ns
Triglycerides	0.998 (0.992–1.004)	0.47	-	ns
Total cholesterol	0.993 (0.988–0.999)	0.03	-	ns
Creatinine	1.015 (0.474–2.173)	0.97	-	ns
Mean heart dose	2.495 (1.936–3.216)	<0.001	2.015 (1.429–2.841)	<0.001
Mean LAD dose	1.257 (1.119–1.412)	<0.001	1.246 (1.04–1.492)	0.02
LAD proximal dose	2.796 (2.017–3.876)	<0.001	1.225 (0.757–1.983)	0.41
LAD middle dose	1.223 (1.099–1.361)	<0.001	1.187 (1.017–1.385)	0.03
LAD distal dose	1.466 (1.271–1.691)	<0.001	1.322 (1.112–1.572)	<0.001

* Calculated using the enter method. ɫ Calculated using the backward Wald method. Abbreviations: BMI, body mass index; DM, diabetes mellitus; HDL, high-density lipoprotein; HR, hazard ratio; HT, hypertension; LDL, low-density lipoprotein; ns, non-significant.

**Table 5:** Kaplan–Meier analysis results.

		Means (95% CI)	Median (95% CI)	*p* *
LDL	<100 mg/dL	44.8 (41.3–48.2) ^a^	44 (39.9–48.1)	<0.001
	100–130 mg/dL	44.8 (42.2–47.4) ^a^	45 (38.7–51.3)	
	130–160 mg/dL	42.6 (40.6–44.5) ^b^	43 (41–45)	
	>160 mg/dL	40.9 (36.6–45.3) ^c^	41 (36.1–45.9)	
	Overall	43.6 (42.2–45)	43 (40.8–45.2)	
DM	Non-diabetic	44.7 (43.1–46.4)	46 (43.8–48.2)	<0.001
	Diabetic	39.9 (37.9–41.9)	39 (36.8–41.2)	
	Overall	43.6 (42.2–45)	43 (40.8–45.2)	
Smoking	Non-smoker	44.1 (42.5–45.7)	44 (41.7–46.3)	<0.001
	Smoker	41.1 (38–44.3)	38 (35.3–40.7)	
	Overall	43.6 (42.2–45)	43 (40.8–45.2)	
Total cholesterol	<160 mg/dL	48.6 (44.9–52.3) ^a^	51 (42.2–59.8)	<0.001
	160–190 mg/dL	48 (45–51.1) ^a^	48 (45–51.1)	
	>190 mg/dL	41.8 (40.3–43.4) ^b^	41 (39.2–42.8)	
	Overall	43.6 (42.2–45)	43 (40.8–45.2)	

* Log rank test is used. **^a–c^** = There is no significant difference between groups with the same letter. Abbreviations: DM, diabetes mellitus; LDL, low-density lipoprotein.

## Data Availability

The data presented in this study are available on request from the corresponding author.

## Abbreviations

ASA: acetylsalicylic acid; BMI, body mass index; CAD, coronary artery disease; CAG, coronary angiography; DM, diabetes mellitus; Hb, hemoglobin; HDL, high-density lipoprotein; HT, hypertension; LAD, left anterior descending artery; LCX, left circumflex artery; LDL, low-density lipoprotein; LMCA, left main coronary artery; Lym, lymphocyte; MPS, myocardial perfusion scintigraphy; RCA, right coronary artery; RT, radiotherapy; WBC, white blood count.
